# Enzymatic Hydrolysis of Water Lentil (Duckweed): An Emerging Source of Proteins for the Production of Antihypertensive Fractions

**DOI:** 10.3390/foods13020323

**Published:** 2024-01-19

**Authors:** Marie-Ève Bernier, Jacinthe Thibodeau, Laurent Bazinet

**Affiliations:** Department of Food Sciences, Laboratoire de Transformation Alimentaire et Procédés ÉlectroMembranaires (LTAPEM, Laboratory of Food Processing and ElectroMembrane Processes), Institute of Nutrition and Functional Foods (INAF), Université Laval, Quebec, QC G1V 0A6, Canada; marie-eve.bernier.7@ulaval.ca (M.-È.B.); jacinthe.thibodeau.1@ulaval.ca (J.T.)

**Keywords:** duckweed, enzymatic hydrolysis, total phenolic content, ACE-inhibitor, bioactive peptide

## Abstract

Water lentil (Duckweed), an emerging protein source, is a small floating aquatic plant with agronomic and compositional characteristics rendering it a potential source of bioactive peptides. However, enzymatic hydrolysis of duckweeds has only been carried out to assess the antioxidant and antimicrobial activities of the hydrolysates. The main objectives of this study were to perform enzymatic hydrolysis of duckweed powder utilizing several enzymes and to evaluate the final antihypertensive activity of the fractions. Duckweed powder was efficiently hydrolyzed by pepsin, chymotrypsin, papain and trypsin, with degree of hydrolysis ranging from 3% to 9%, even without prior extraction and concentration of proteins. A total of 485 peptide sequences were identified in the hydrolysates and only 51 were common to two or three hydrolysates. It appeared that phenolic compounds were released through enzymatic hydrolyses and primarily found in the supernatants after centrifugation at concentrations up to 11 mg gallic acid/g sample. The chymotryptic final hydrolysate, the chymotryptic supernatant and the papain supernatant increased the ACE inhibitory activity by more than 6- to 8-folds, resulting in IC_50_ values ranging between 0.55 to 0.70 mg peptides/mL. Depending on the fraction, the ACE-inhibition was attributed to either bioactive peptides, phenolic compounds or a synergistic effect of both. To the best of our knowledge, this was the first study to investigate the enzymatic hydrolysis of duckweed proteins to produce bioactive peptides with therapeutic applications in mind.

## 1. Introduction

Hypertension, also known as high or raised blood pressure, has been recognized as one of the leading causes of premature death worldwide [1,2]. Uncontrolled hypertension stands as a significant risk factor for cardiovascular disease responsible for 17.9 million deaths annually [3]. Lifestyle changes such as adopting a healthy, low-salt diet, engaging in exercise and quitting smoking can help reduce hypertension. However, for certain individuals, lifestyle adjustments prove insufficient, necessitating medical treatment for the condition. However, hypertension medication carries a range of undesired side effects, including dry cough, dysgeusia and skin rashes [1], hence the importance of exploring alternative solutions. In addition to their minimal environmental impact and compelling nutritional properties, the consumption of plant proteins has demonstrated beneficial effects on human health, notably in reducing the risk of cardiovascular disease [4]. Several compounds within plant matrices underlie these positive biological effects, among them bioactive peptides [5]. Consequently, the consumption of these protein fragments, whether alone or incorporated into food formulations, has emerged as a promising avenue for natural alternative to synthetic drugs in the realm of chronic diseases prevention and long-term health and wellness [6].

Angiotensin-converting enzyme (ACE) plays a significant role in the regulation of blood pressure, converting angiotensin I into the potent vasoconstrictor angiotensin II while also breaking down the vasodilator bradykinin, leading to an elevation in blood pressure [7]. Antihypertensive peptides work mainly by inhibiting ACE, effectively reducing blood pressure levels [8]. Several plant protein sources have undergone investigations to assess their potential for generating ACE inhibitors [9], including soy proteins [10], rapeseed proteins [11], flaxseed proteins [12], amaranth leaf proteins [13] and alfalfa proteins [1,14].

Duckweeds, small floating aquatic plants, are still relatively unfamiliar in Western nations, despite their extensive historical consumption in Asian cultures [15]. The primary focus of research and industrial applications revolved around its utilization in water treatment, as a biofuel or for animal nutrition purposes [16]. While an increasing array of studies is delving into their potential for human nutrition [15,17,18], only a limited number of clinical studies have explored the link between duckweed consumption and its effects on human health [19,20], and the specific compounds responsible for these effects have received scant attention so far [21,22]. Duckweed boasts a substantial crude protein content, reaching up to 40% on a dry basis and displays rapid growth (biomass doubling within 24 to 48 h) [18,23], rendering it a captivating candidate as a potential source of bioactive peptides.

Enzymatic hydrolysis stands as the most widely employed method for producing bioactive peptides and constitutes a key step demanding meticulous control to generate hydrolysates imbued with bioactive potential [8,24]. Plant proteins serve as robust reservoirs of biologically active peptides and have prompted a considerable number of studies exploring the bioactivities spanning an extensive array of plant matrices [5,8,25]. Nonetheless, to the best of our knowledge, enzymatic hydrolysis of duckweeds has been carried out only twice to date [26,27], with these endeavors directed towards assessing the antioxidant, functional and antimicrobial attributes of the resulting hydrolysates. However, these studies did not characterize the peptide sequences present, and no studies reported the antihypertensive activities of duckweed hydrolysates.

In this context, a novel plant protein source, duckweed, underwent hydrolysis to yield potential antihypertensive peptides. The specific objectives of this project were: (1) to perform enzymatic hydrolysis of duckweed powder utilizing several enzymes (pepsin, chymotrypsin, papain and trypsin) and evaluate the degree of hydrolysis (DH) of the different hydrolysates; (2) to assess the impact of hydrolysate centrifugation by characterizing the fractions obtained and identifying the peptide sequences present; (3) to evaluate the total phenolic content (TPC) of each fraction before and after centrifugation of the hydrolysates, and (4) to evaluate ACE-inhibitory activity (antihypertensive activity) of the generated fractions.

## 2. Materials and Methods

### 2.1. Materials

#### 2.1.1. Duckweed Powder

Defatted duckweed powder was purchased from Seta organic (Quebec, Canada). The proximal composition of the powder was previously described by Muller et al. [28] and consisted of 40.7% soluble fibers, 6.5% insoluble fibers, 6.2% ashes and 3.7% moisture. The protein content was approximately 38.6%.

#### 2.1.2. Enzymes for Hydrolysis

Pepsin from porcine gastric mucosa (lyophilized powder, ≥3200 units/mg protein, lot # SLCH7086), α-Chymotrypsin from bovine pancreas (lyophilized powder, ≥40 units/mg protein, lot # SLCH1926), Papain from papaya latex (≥8.0 units/mg protein, lot # SLCJ3270) and Trypsin from bovine pancreas (powder, ≥7500 BAEE units/mg solid, lot # SLCM7280) were purchased from Sigma-Aldrich (Oakville, ON, Canada). Here, papain was chosen since it comes from a vegetable to fully meet expectations of vegan or vegetarian people.

#### 2.1.3. Chemicals

Hydrochloric acid (HCl) and sodium hydroxide (NaOH), used to maintained pH during hydrolyses, were purchased from Fisher Scientific (Montreal, QC, Canada). The reagents used for determination of the degree of hydrolysis were sodium tetraborate (Merck, Darmstadt, Germany), sodium dodecyl sulfate (SDS) (Bio-Rad Laboratories, Ontario, Canada), o-phthaldialdehyde (OPA) (Sigma Aldrich, St. Louis, MO, USA), beta-mercaptoethanol (Sigma Aldrich, St. Louis, MO, USA) and D-L-Leucine (Sigma Aldrich, St. Louis, MO, USA). Methanol used for polyphenol extraction was purchased from Fisher chemical (Ottawa, ON, Canada). The reagents used for the detection of phenolic compounds were Folin-Ciocalteu (Sigma Aldrich, St. Louis, MO, USA), sodium carbonate (Merck, Darmstadt, Germany) and gallic acid (Sigma Aldrich, St. Louis, MO, USA). UPLC chemicals (LC-MS grade water, LC-MS grade acetonitrile and formic acid) were purchased from Fisher (Ottawa, ON, Canada). For ACE inhibitor analysis, borax (Merck, Darmstadt, Germany), boric acid (Anachemia, Montréal, QC, Canada), NaCl (Fisher, Ottawa, ON, Canada), phosphate (Anachemia, Winnipeg, MB, Canada), TT reagent (cyanuric chloride from Fisher scientific, Ottawa, ON, Canada and 1,4-dioxane from Merck, Darmstadt, Germany), HHL (powder, ≥98% (HPLC), Sigma Aldrich, St. Louis, MO, USA), enalapril (Thermo scientific, MA, USA) and Angiotensin-converting enzyme (ACE) (Sigma Aldrich, St. Louis, MO, USA) were used.

### 2.2. Enzymatic Hydrolysis of Duckweed Proteins

#### 2.2.1. Preparation of Duckweed Solution Prior to Hydrolysis

**Figure 1:**
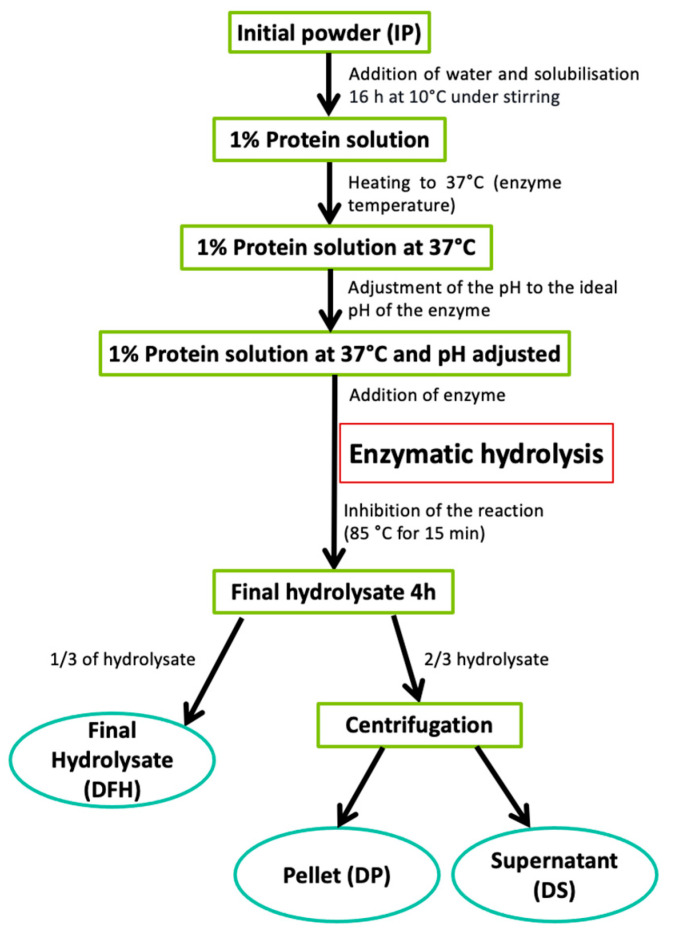
Protocol used for enzymatic hydrolysis and production of the three fractions.

Duckweed powder (2.79 g) was suspended in 97.41 mL of distilled water (1% proteins *w*/*w*) and left to solubilize 16 h at 10 °C under constant stirring (Figure 1). The solutions were heated to 37 °C, the temperature for hydrolysis. Then the solutions were adjusted to the specific pH of each enzyme, and the enzyme was added to begin the 4 h-hydrolysis. In this study, enzymatic hydrolysis of proteins was performed on duckweed powder directly, i.e., duckweed that was dried, defatted and then grounded. No process was performed to extract the proteins in the product beforehand.

#### 2.2.2. Duckweed Hydrolysis Protocol

The duckweed solution was hydrolyzed using four proteases: pepsin, chymotrypsin, papain, and trypsin. Proteases were selected based on the literature, which reported in vitro [29,30] or in silico [31,32,33] successful recovery of bioactive peptides by enzymatic hydrolysis of plant proteins similar to water lentils. These proteases were added to the duckweed solution at a ratio of 1:100 (enzyme:substrate). The hydrolysis conditions used for the experiment were 37 °C, pH 2 for pepsin [34], pH 6.5 for papain [35] and pH 8 for trypsin [34] and chymotrypsin [34]. During the 4 h-hydrolysis, the pH was maintained using either 1 M NaOH or 1 M HCl and the temperature was stabilized using a hot plate with thermometer included (VWR, Montreal, Canada). Samples were taken at 0, 30, 60, 120 and 240 min and filtered through a 0.45 µm PTFE filter for the assessment of the DH. After 4 h of hydrolysis, the inactivation of enzymes was carried out by heating the solutions at 85 °C for 15 min. After enzyme inactivation, 1/3 of the volume of the hydrolysate was frozen while 2/3 of the hydrolysate were centrifuged (9000 × *g* for 30 min at 4 °C) to obtain a pellet and a supernatant before freezing. This allowed to obtain three fractions following hydrolysis: Duckweed final hydrolysate (DFH), duckweed pellet (DP) and duckweed supernatant (DS) (Figure 1). This separation was carried out with the aim to potentially valorize all fractions resulting from hydrolysis or to demonstrate if the hydrolysate of the entire product is sufficient to produce a bioactive fraction with no further separation step in a circular economy approach [36]. Currently, in the literature, only supernatants are analyzed and valorized, while the pellets are discarded. Each fraction was then freeze-dried and stored at −20 °C for future analyses. Thus, this protocol led to 12 different fractions following hydrolysis (four enzymes and three different fractions per enzyme). The hydrolyses were performed in triplicate. The 12 different fractions were analyzed in terms of DH, total nitrogen content, reverse-phase ultra-high pressure liquid chromatography (RP-UPLC) and mass spectrometry (MS) analyses, TPC and antihypertensive activity.

### 2.3. Analyses

#### 2.3.1. Measurement of the Degree of Hydrolysis (DH)

The DH representing the number of cleaved peptide bonds in relation to time of hydrolysis was evaluated using the ortho-phtaldialdehyde (OPA) method described previously [37] with slight modifications. Briefly, 0.160 g of solid OPA was diluted in 4 mL methanol, then mixed with 100 mL of 100 mM tetraborate, 10 mL of 20% SDS and 400 uL β-mercaptoethanol to form OPA reagent. D-L-leucine diluted at different concentrations (0.00, 0.75, 1.5, 2.25 and 3.0 mM) with SDS 1% was used as a standard to generate a calibration curve. To carry out the reaction of o-phtaldialdehyde and β-mercaptoethanol with α-amino groups released during hydrolysis, 3 mL of OPA reagent were in contact with 150 µL of standard or diluted samples for two minutes at room temperature. The proportion of cleaved bonds was quantified by spectrophotometry at 340 ƞm (HP8453, Agilent Technologies, Santa Clara, CA, USA) and compared with the total α-amino group content of the sample. Acidic hydrolysis of duckweed was performed in 6 M HCl at 110 °C for 24 h [38] in a Pierce Reacti-Therm Heating module (Pierce chemical company, box 117, Rockford, IL 61105) to determine total α-amino group content (h_tot_), since this data specifically for duckweed was not reported in the literature, and assuming that the proteins underwent complete hydrolysis of all peptide bonds during acid digestion. The h_tot_ obtained was 8.7 ± 0.40 meq/g of protein. This value of total amount of peptide bonds in the protein substrate was similar to those obtained by other researchers on mulberry leaf protein which was 8.16 [39] and on defatted wheat germ globulin with a value of 7.64 [40]. Also, according to Nielsen et al. [41], the value of h_tot_ for most proteins is about 8, which is consistent with the value obtained in this study. In addition, the value obtained is comparable to the values found by Adler-Nissen for other plant proteins (values between 7.8 and 9.2 depending on the source of vegetable protein) [42], which is an established reference used to get this information [43,44]. The following equation was used to calculate ***DH***:

$$DH=\frac{\left(h-h0\right)}{htot}\times 100$$

where ***DH*** is the degree of hydrolysis (%), ***h*** the number of hydrolyzed peptide bonds during the enzymatic hydrolysis of duckweed proteins (meq/g protein), ***h*_0_** the number of hydrolyzed peptide bonds before the addition of enzyme (meq/g protein), and ***h_tot_*** the number of total peptide bonds in duckweed powder determined as previously mentioned (8.7 meq/g protein). Three repetitions of hydrolysis for each enzyme were performed and the DHs presented were averaged values from these three repetitions.

#### 2.3.2. Total Nitrogen Content and Protein/Peptide Recovery Yield of the Fractions

The total nitrogen content of the fractions was evaluated by the Dumas combustion method using a Rapid Micro N Cube (Elementar Analysensysteme GmbH, Langenselbold, Germany). The protein/peptide content was calculated from nitrogen content with 6.25 as the conversion factor from nitrogen to protein as commonly used for leaf proteins [17,45,46].

Then, after centrifugation, the protein/peptide yield (%) in the supernatant and pellet was calculated using the mass of powders recovered following freeze-drying and the protein/peptide content of the fractions. The protein/peptide yields of pellets and supernatants were calculated with the following equation:

$$protein/peptide\;recovery\;yield\;(\%)=\frac{pfa*ma}{pft*mt}\times 100$$

where ***pf_a_*** is the protein/peptide content of the fraction (DP or DS fraction), ***m_a_*** the mass of powder obtained after freeze-drying for the fraction (DP or DS fraction), ***pf_t_*** the protein/peptide content for the final hydrolysate (DFH fraction) and m_t_ the total mass of powder obtained after freeze-drying (DP and DS combined only).

#### 2.3.3. RP-UPLC and MS Analyses

RP-UPLC and MS analyses were performed according to the method described by Cournoyer et al. [47] with slight modifications. Briefly, analyses were performed using a 1290 Infinity II UPLC (Agilent Technologies, Santa Clara, CA, USA) consisting of a binary pump, a multisampler, an in-line degasser and a variable wavelength detector adjusted to 214 nm. Final hydrolysate, supernatant or pellet samples prepared at 1% protein (m/m) were filtered (0.45 µm PVDF filter) into a glass vial and loaded (3 µL) onto an InfinityLab Poroshell 120 SB-AQ column (2.1 × 100 mm, 2.7 micron, Agilent, Santa Clara, CA, USA). The column temperature was set at 45 °C, a flow rate of 400 µL/min was applied with a maximum pressure of 600 bar. A gradient consisting of solvent A (LC-MS grade water with 0.1% formic acid) and solvent B (LC-MS grade acetonitrile with 0.1% formic acid) was implemented from 0% B to 45% B in 30 min. Then, ramping to 95% B in 15 min and maintained for 5 more min. Finally, back to initial conditions for 5 min before the next injection.

The compounds present in the hydrolysates were determined using Agilent’s 6560 hybrid ion mobility quadrupole time-of-flight mass spectrometer ((IM-Q-TOF), Santa Clara, CA, USA). All LC-MS/MS experiments were acquired using Q-TOF. Signals were recorded in positive mode at Extended Dynamic Range, 2 Ghz, 3200 *m*/*z* with a scan range between 100–3200 *m*/*z*. Nitrogen was used as the drying gas at 13.0 L/min and 150 °C, and as nebulizer gas at 30 psi. The capillary voltage was set at 3500 V, the nozzle voltage at 300 V and the fragmentor at 400 V. The system was calibrated using an ESI-L low concentration tuning mix (Agilent, Santa Clara, CA, USA). Data acquisition and analysis was done using the Agilent Mass Hunter Software package (LC/MS Data Acquisition, Version B.09.00, Qualitative Analysis, Version B.07.00 Service Pack 2 including BioConfirm Software). Duckweed (Lemna) with Taxonomy ID 4469 database containing 28,643 entries from NCBI (duckweed-Protein-NCBI (nih.gov), accessed date: 15 September 2022) was downloaded to be used in Spectrum Mill MS Proteomics Workbench Software (Version B.06.00) to perform peptide sequence identification.

#### 2.3.4. Determination of Total Phenolic Content (TPC)

##### Extraction of Phenolic Compounds

DFH, DP, DS and IP were prepared at a concentration of 25 mg of powder/mL with methanol 80% as solvent [48,49]. Then, the extraction was carried out at 50 °C [46] in an ultrasonic bath for 60 min in 5 mL microcentrifuge tubes and samples were vortexed every 10 min. After one hour bath extraction, the samples were vortexed and filtered through 0.45 µm PTFE filter [21] to separate the insoluble part from the soluble part. The filtrates of the samples were collected in 2 mL microcentrifuge tubes for the total phenolic content analysis.

##### Total Phenolic Content Determination

The determination of TPC was carried out using the Folin-Ciocalteu method [48]. Gallic acid was used as a standard and was prepared at concentrations of 0.0, 2.5, 5, 10, 25, 50, 100, 250 and 500 mg/L to obtain a standard curve. For the reaction, 20 µL of distilled water, samples or standard solutions were added to the wells of a 96-well microplate. Then 100 µL of 1:10 diluted Folin-Ciocalteu was added to the wells. An incubation period of 4 min was necessary for the oxidation of the phenolic compounds by the Folin reagent. Afterwards, 80 µL of 7.5% sodium carbonate was added to inactivate the reaction. The microplate was incubated in the microplate reader for 45 min, and the absorbance was measured at 765 ƞm with an xMark Microplate spectrophotometer (Bio-Rad, Mississauga, ON, Canada). TPC was expressed as mg gallic acid equivalent/g sample.

#### 2.3.5. Evaluation of Antihypertensive Activity

##### Evaluation of ACE-Inhibition of Fractions In Vitro

The enzymatic hydrolysis of leaf protein yields peptides that exhibit various biological activities, including antioxidant, opioid, antihypertensive, antibacterial, antinociceptive, and memory-enhancing effects [6,50]. Being one the most studied biological activities [8], the focus in this study was placed on investigating the antihypertensive activity of fractions obtained by assessing ACE inhibition in vitro.

The antihypertensive capacity was evaluated using a spectrophotometric ACE-inhibitory assay, originally developed by Hayakari et al. [51], with minor adjustments [52]. Samples were prepared at 0.5–4.0 mg peptide/mL for DFH and DP and at 0.125–2.0 mg peptide/mL for DS based on preliminary tests and to ascertain the IC_50_ values. Enalapril was used as positive control, while water served as blank [52]. Briefly, 20 µL of sample, enalapril or blank was combined with 20 µL of enzyme at a concentration of 0.25 U/mL prepared in a pH 8.3 borate buffer (composed of 4.05 g H_3_BO_3_, 4.86 g KCl, 63.2 g NaCl, and NaOH 1 M to adjust the pH in 1 L). Additionally, 80 μL of pH 8.3 phosphate buffer (consisting of 27.2 g KH_2_PO_4_ and NaOH 1 M to adjust the pH in 1 L) was added. The mixture was then vortexed and centrifuged for 10 s each. Next, the mixture (120 μL) was incubated for 10 min at 37 °C, while an identical sample set underwent incubation at 95 °C for 10 min to deactivate ACE and serve as negative control. After cooling on ice, each tube was supplemented with 40 μL of N-Hippuryl-His-Leu-Hydrate (HHL) (6.25 mM in borate buffer), vortexed and centrifuged for 10 s each. Subsequently, the tubes were incubated for 60 min at 37 °C. Following another 10-min incubation at 95 °C, each tube received 480 μL of pH 8.3 phosphate buffer and 360 μL of chlorure cyanurique (TT) reagent 3% (30 mg/mL in 1,4-dioxane). The mixture was vortexed and centrifuged again for 10 s each, and, after these steps, 200 μL of the supernatant was pipetted into a 96-well microplate. The absorbance was measured at 382 nm using an xMark^TM^ spectrophotometer (Bio-Rad, Mississauga, ON, Canada) and the calculation of ACE inhibition was performed using the following equation [53]:

$$\mathrm{ACE}\mathrm{inhibition}\;(\%)=\frac{\left(Ec-Es\right)}{\left(Ec-Eb\right)}\times 100$$

where ***Ec*** is the absorbance of the reaction mixture with water (blank), ***Es*** the absorbance of the reaction mixture with sample to test and ***Eb*** the absorbance of the reaction mixture with sample to test but heated to 95 °C at the beginning of the experiment (negative control) [52].

##### Screening for Potential Antihypertensive Sequences

To push our research further, among the peptide sequences identified, those common to more than two hydrolysates were verified as potential antihypertensive in AHTPDB (https://webs.iiitd.edu.in/raghava/ahtpdb/pepsearch.php) (accessed on 12 August 2023).

#### 2.3.6. Statistical Analyses

##### UPLC-MS/MS Data Treatment

UPLC-MS/MS data treatment were processed using Profinder software (Version 10.0) and Mass Profiler Professional (Version 15.1) to perform statistical analysis on the data obtained under various conditions, based on the ion abondance of each compound, as previously conducted by Cournoyer et al. [47]. Heatmaps and principal component analysis (PCA) were generated following the filtering of all entities by frequency. The remaining compounds where then subjected to a non-parametric Kruskal-Wallis test for comparing the average ion intensity across conditions [54]. This test was chosen for its reliability and robustness, as the assumptions of normality and variance homogeneity were not strongly demonstrated [47]. Notably, the Kruskal-Wallis was suitable irrespective of the data distribution [55]. To control the type I error rate, the false discovery rate (FDR) method, also known as the Benjamini-Hochberg FDR method, was employed to identify which peptides are different between conditions [56]. Corrected *p*-values were obtained using a permutation resampling version of this method, involving 10,000 permutations [47]. Subsequently, for the significant peptides, the Multiple Comparison Tukey HSD test was applied to identify which conditions were different from others [47,57]. A hierarchical cluster heatmap and a PCA were then performed on the remaining peptides, aiming to identify potential peptide grouping and analyze the overall behavior of the conditions. During this analysis, the peptides were log2 transformed to approximate normality in the factorial scores [47].

##### Other Statistical Analyses

All experiments were conducted with three independent samples and the values were reported as mean ± standard deviation. Statistical analyses were performed using SigmaPlot software (version 14.0, Systat Software, San Jose, CA, USA). One-way analyses of variance (ANOVA) were performed to compare the DH, the purities, the protein/peptide yields, the TPC and the bioactivities. Statistical differences between fractions were analyzed by Tukey test (*p* < 0.05). The Tukey tests were carried out on the various fractions (DFHs, DPs, DSs) obtained with an enzyme as well as on the same fraction obtained with the various enzymes. For protein/peptide yields and total phenolic contents, *t*-tests were also performed to compare pellet (DP) to supernatant (DS), and each fraction to the initial powder (IP), respectively.

## 3. Results

### 3.1. Degree of Hydrolysis (DH)

**Figure 2:**
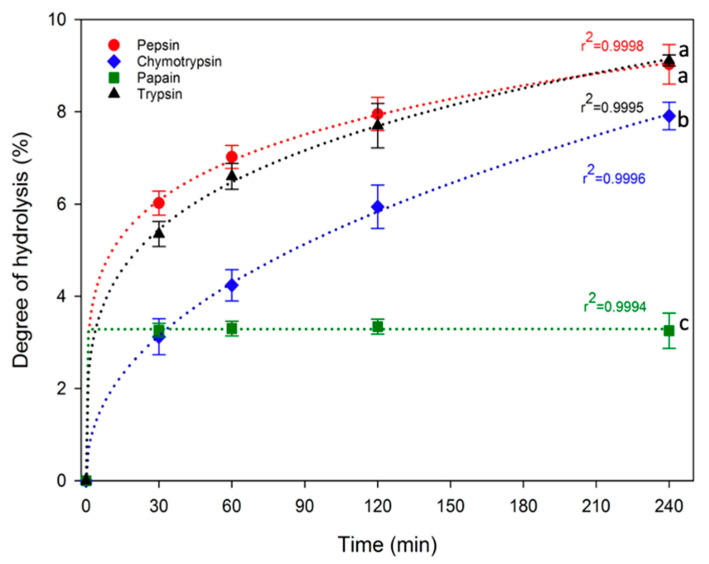
Evolution of DH during enzymatic hydrolysis of duckweed powder (initial powder: IP) by pepsin, chymotrypsin, papain and trypsin. For values at t = 240 min., values with different lowercase letters are significantly different, according to one-way ANOVA *p* < 0.05 (Tukey test) and error bars represent the standard errors.

In this study, enzymatic hydrolysis of proteins was conducted directly on duckweed powder, specifically dried, defatted and, subsequently, grounded duckweeds. No additional process was undertaken to extract the proteins from the product to keep the process as simple as possible. The DH values for pepsin, chymotrypsin, papain and trypsin duckweed protein hydrolysates at 0, 30, 60, 120 and 240 min are shown in Figure 2. There was a rapid hydrolysis of the protein in the first 30 min for all enzymes used leading to 3.12–6.02% DH for the hydrolysates. After that time, DH increased progressively up to 240 min for all enzymes except for papain which did not increase further after 30 min. At the end of protein hydrolysis, pepsin and trypsin produced the highest DH (9.03 ± 0.43% and 9.11 ± 0.12%, respectively), followed by chymotrypsin (7.91 ± 0.30%), while papain hydrolysis resulted in the lowest DH (3.25 ± 0.38%). The enzymatic hydrolyses follow power law (y = ax^b^), displaying high R^2^ values ranging from 0.9994 to 0.9998.

**Figure 3:**
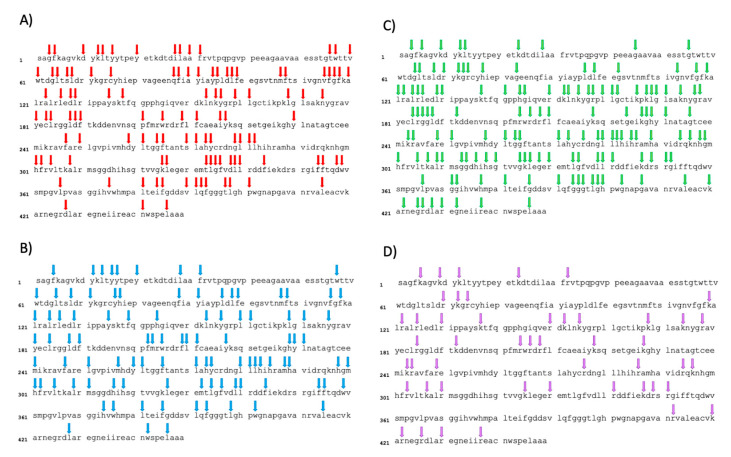
Theoretical cleavage sites of ribulose-1,5-biphosphate carboxylase, partial (chloroplast) [*Lemna gibba*] (ncbi, protein, 449 AA, ACCESSION AAK72524, VERSION AAK72524.1) by (**A**) Pepsin; (**B**) Chymotrypsin; (**C**) Papain and (**D**) Trypsin. Cleavage occurs at the right of the arrow. For pepsin, chymotrypsin and trypsin, PeptideCutter (https://web.expasy.org/peptide_cutter/, accessed on 16 August 2023) was used to identify cleavage sites. For papain, the theoretical cleavage sites were identified using the supplier’s instructions (https://www.sigmaaldrich.com/CA/en/product/sigma/p5306, accessed on 16 August 2023), since the enzyme wasn’t available on PeptideCutter.

The assessment of the degree of hydrolysis allowed to determine the efficiency of hydrolysis and represented the number of peptide bonds cleaved within the proteins [34]. Despite the relatively low protein content of the utilized duckweed powder (approximately 40%) compared to a protein concentrate or isolate traditionally used for enzymatic hydrolysis, along with the presence of other potentially interfering compounds such as fibers [58], the hydrolysis appeared to have been successful, demonstrated by consistent degrees of hydrolysis in this study. The differences in the degrees of hydrolysis can be explained by the specific cleavage sites targeted by each enzyme selected for this study. With the intention of elucidating DH results in relation to the cleavage sites targeted by the diverse enzymes employed, Figure 3 illustrated the sequence of ribulose-1,5-biphosphate carboxylase, partial (chloroplast) [*Lemna gibba*] (ncbi, protein, 449 AA, ACCESSION AAK72524, VERSION AAK72524.1) along with the theoretical cleavage sites associated with the various enzymes. This segment of the protein was selected since a significant portion of the identified peptides within each of the four final hydrolysates (DFHs) stems from this sequence. Pepsin, characterized by broad specificity, cleaves between two aromatic amino acids (AA), between an aromatic AA and a dicarboxylic AA and on the carboxylic side of phenylalanine and leucine [9]. It has 112 theoretical cleavage sites for this protein, which is consistent with the rather high DH. Chymotrypsin, on the other hand, cleaves the C-terminal side of aromatic amino acids, leucine and methionine [59]. It presents 100 theoretical cleavage sites within the protein (slightly less than pepsin), corroborating the slightly lower DH observed. Papain operated by cleaving the carboxyl side of basic amino acids, leucine or glycine, unless these amino acids are succeeded by valine [60]. Despite the theoretical presence of 141 cleavage sites in the chosen protein, seemingly at odds with the low DH, an examination of the proportion of sequences originating from this protein for each enzyme (as depicted in Table 1) shed light on this discrepancy. Notably, for papain hydrolysate, a higher proportion of sequences (~42%) stems from this protein, compared to approximately 31% and 36% for pepsin and chymotrypsin, respectively. This observation implied that papain tends to target RuBisCO proteins more than others, leading to a high number of cleavage sites within this protein and yielding to a low DH at the same time. Trypsin, distinguished by its high specificity, cleaves on the carboxyl side of lysine or arginine, unless succeeded by proline [59]. This results in 48 theoretical cleavage sites within the protein, seemingly contradicting the high DH. However, upon assessing the proportion of sequences originating from this protein for each enzyme (Table 1), a distinct trend emerged. Unlike papain, the trypsin hydrolysate comprised a lower proportion of sequences (~23%) originating from this protein. This indicated that trypsin exhibited lesser potential for cleaving RuBisCO proteins and instead targeted other duckweed proteins more efficiently. Overall, the higher DH for pepsin and trypsin demonstrated the presence of peptide bounds within duckweed proteins that were more susceptible to hydrolysis compared to chymotrypsin and papain [34]. However, it is important to keep in mind that a higher degree of hydrolysis does not necessarily mean a more biologically interesting hydrolysate.

To our knowledge, enzymatic hydrolysis of duckweed proteins has only been performed twice on duckweed proteins powder [26,27]. In the first study, Tran et al. performed enzymatic hydrolyses of proteins from defatted *Lemna minor* using flavourzyme and alcalase at different concentrations and times and they evaluated the antioxidant property of the hydrolysates. The DHs obtained varied between 9.45% and 16.34% [26], which is significantly higher than those obtained in the present study. These differences can be explained by different factors: the enzymes used for hydrolyses were not specific, the raw material was different (blend of several duckweed species from Florida vs. *Lemna minor* from Vietnam), differences in hydrolysis conditions (higher temperature, different durations and enzyme concentrations), and the methods used to measure the DH were different (OPA vs. TNBS assay). In the second study, Duangjarus et al. used alcalase to hydrolyze duckweed (*Wolfia globosa*) proteins and they investigated antimicrobial and functional properties of the fractions obtained [27]. Unfortunately, they also used a non-specific enzyme for hydrolysis, and they did not measure the DH.

On another source of leaf proteins, Famuwagun et al. hydrolyzed eggplant leaf proteins using pepsin, trypsin, and chymotrypsin with similar hydrolysis conditions to those used in this study and using the same method for DH evaluation. The DHs obtained by the researchers (between 18 and 30%) are significantly higher than those obtained in the present study [34]. This can be explained by the fact that they performed an additional step of chemical extraction of the proteins to produce a protein isolate on which they then did the enzymatic hydrolysis. This step allowed them to concentrate proteins up to 80% in their product and remove unwanted compounds, such as insoluble proteins and fibers, but they also lost several compounds with the potential to be valorized. Also, it is interesting to note that trypsin was the enzyme that led to the highest DH as in the present study. Using pepsin and papain as in the present study, Schlegel et al. hydrolyzed a lupin protein isolate and obtained DHs of 2.61 for papain and 3.37 for pepsin [43]. The DH obtained with papain was similar to the one obtained in this study while the one obtained with pepsin was lower, which again can be explained by differences in the hydrolyzed proteins (leaf vs. legume) as well as the hydrolysis conditions (higher temperature, lower E/S ratios and lower hydrolysis duration in their study).

### 3.2. Protein/Peptide Content and Protein/Peptide Recovery Yield of the Fractions

The protein/peptide content and protein/peptide yield of the different fractions are shown in Table 2. Regardless of the enzyme used, the protein/peptide content of the supernatants were always higher than that of the pellets and the complete fractions. For papain, there were no significant differences between the protein/peptide content of DFH and DP, whereas for the other enzymes, the protein/peptide content of DFH were always higher than the protein/peptide content of DP. For DS fractions, the highest protein/peptide content was obtained with trypsin (67.77%), followed by chymotrypsin (64.73%), papain (56.93%) and then pepsin (50.12%). For the DP fractions, the highest protein/peptide content was obtained with papain (34,84%), significatively higher than the ones obtained with chymotrypsin (27.02%) and pepsin (26.34%). Trypsin pellet protein/peptide content (23.86%) was not significatively different from pepsin (26.34%) and both enzymes led to the lowest protein/peptide content.

Protein/peptide yield was higher in the DS fraction for pepsin and trypsin, while it was higher in the DP fraction for papain. For chymotrypsin, there was no significant difference between DP and DS recovery yields (Table 2).

Enzymatic hydrolysis of proteins allowed the release of peptides into the final hydrolysate. As peptides are soluble, they were recovered mainly in the supernatant of the fractions [61], which would explain why the supernatants had a higher protein/peptide content than the pellets and the final hydrolysates. We might be inclined to believe that the higher the DH, the higher the protein content of the supernatant would be. This is indeed the case for the supernatants of hydrolysates whose hydrolysis was performed around neutral pH (with chymotrypsin, papain and trypsin) because at this pH, some proteins are insoluble (ending up in the pellet) and others, as well as peptides, are soluble (being recovered in the supernatant). However, for the peptic hydrolysate, as the hydrolysis and centrifugation occurred at acidic pH (pH 2), protein solubility was very low, which means that the majority of proteins were insoluble and, therefore, collected in the pellet, and only peptides were in the supernatant, thus depleting the supernatant in proteins and peptides [62]. Also, there was a direct link between protein/peptide recovery yield and DH: For hydrolyses with higher DH (pepsin and trypsin), the protein/peptide recovery yields were higher in DS, for hydrolysis with the lowest DH (papain), the protein/peptide recovery yield was higher in DP, while for the hydrolysis with intermediate DH (chymotrypsin), there was no significant difference between protein/peptide recovery yields of DS and DP.

### 3.3. Characterization and Identification of Peptides by UPLC-MS/MS

#### 3.3.1. Molecular Weight Distribution of Peptides in Each Fraction

**Figure 4:**
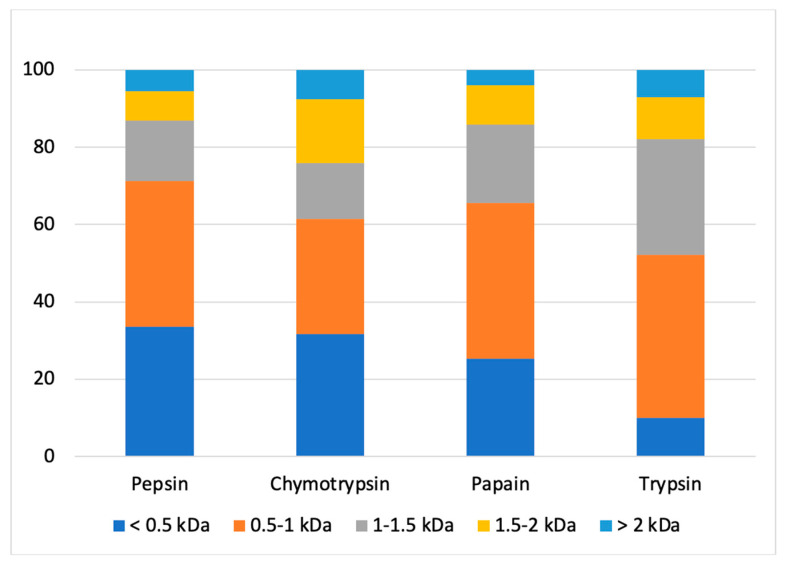
Peptide molecular weight distribution in the fractions obtained after 240 min of hydrolysis with the four enzymes tested.

Following the injection of samples on UPLC-MS/MS, the compounds contained in each fraction were identified. After statistical analysis, 1327 compounds were found in all hydrolysates combined. After comparison with the peptide sequence lists generated by Spectrum Mill and using duckweed database for the different enzymes, a total of 485 peptides were matched (retention time, mass, and sequence). Sequences from bacterial proteins in the duckweed database were not considered. The molecular weight distribution of peptides in each fraction (Figure 4) showed that peptic, chymotryptic and papain hydrolysates contained mainly low molecular weight (MW) peptides (<1 kDa) for between 60 and 70% approximately of the peptides contained in the fraction. These results were similar to a previous work on alfalfa leaf protein hydrolysate which reported 67.86% of <1 kDa peptides of a hydrolysate obtained with alcalase [63]. On the other hand, the hydrolysate obtained with trypsin contains 50% peptides with a MW lower than 1 kDa and 50% peptides with a MW higher than 1 kDa. More precisely, the fraction generated by trypsin had a different molecular weight distribution than those generated with the other enzymes, namely a lower percentage of compounds with molecular weights inferior to 0.5 kDa and a higher percentage of compounds with molecular weights between 1 and 1.5 kDa than the other fractions. These results suggested that duckweed protein cleavage sites by trypsin led to the formation of peptides with higher molecular weights. Similar to what was done in the present study, Sun et al. studied molecular weight distributions of mulberry leaf protein hydrolysates. They used six enzymes for enzymatic hydrolysis (alcalase, protamex, papain, flavourzyme, neutrase and trypsin) and two of these enzymes were common to our study. As in the present study, the authors found that hydrolysis with trypsin led to the formation of peptides with higher MW than other fractions, explained by FTIR analysis which showed that trypsin could not completely destroy the protein structure of mulberry leaf protein [39]. Finally, Figure 4 showed that the chymotryptic hydrolysate had higher relative abondance of peptides with molecular mass over 1.5 kDa, more specifically those with a molecular mass between 1.5–2 kDa. These results suggest that duckweed protein cleavage sites by chymotrypsin also lead to the formation of peptides with high MWs.

The results revealed that the hydrolysates exhibited different peptide molecular weight distributions, which align with the variation in protease specificity among the enzymes used. This observation was also made by other research teams on other leaf protein hydrolysates [39,64]. This confirmed that the hydrolysis was effective and efficient to produce low MWs peptides but also, in some cases, peptides with high MWs. The fluctuations in molecular weight distribution of the hydrolysates, due to the chosen enzymes, can be utilized to anticipate the bioactive properties of the hydrolysates.

#### 3.3.2. Heat Map

**Figure 5:**
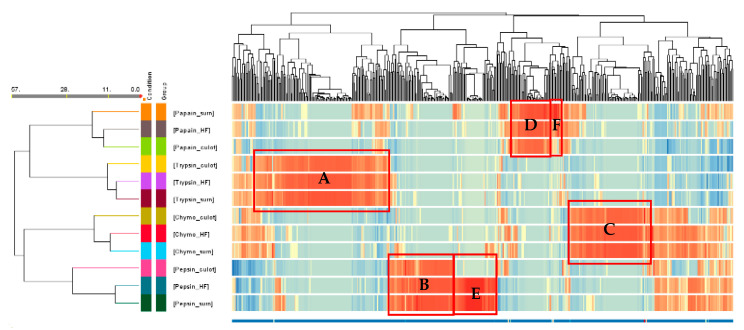
Hierarchical cluster on hydrolysis conditions, type of fraction and peptides (*n* = 485), similarities measured with Euclidean distance and heatmap.

A hierarchical cluster analysis was conducted, involving the generation of a heatmap and utilizing Euclidean distance (Figure 5). This analysis was performed by averaging the MS/MS ion abundance data for each peptide across all conditions, as previously done by Cournoyer et al. [47]. In this figure, each peptide is represented as a function of its normalized ion abundance. The software automatically generated a range for the color scale based on this normalized ion abundance parameter and compared it to the same peptides in the different fractions and between peptides in the same fraction. The darker the red sequences, the more present they were in the fractions and relative to the other fractions, while the darker the blue sequences, the less they were present in the fractions. This figure allows to depict at one sight the variations and similarities in the peptide composition among the retrieved fractions under each condition (enzyme and fraction).

The cluster on the hydrolysis conditions and type of fraction showed 11 clustering nodes. The Euclidean distance between the fractions is also shown on the left. Since the first node represents the most similar conditions based on the Euclidean distance, it was possible to notice that, for all enzymes, the supernatant was the fraction that was the most similar to the final hydrolysate in terms of peptide population for the same enzyme, except for papain which was the pellet. Then, the fraction that was to the most different to the other two fractions from the hydrolysis with the same enzyme was the pepsin pellet. It also appeared that there were differences in peptide populations between fractions obtained with different enzymes. Indeed, some groups of peptides stood out (boxed in red in Figure 5), since they were upregulated in the 3 fractions generated by the same enzyme. Thus, these peptides represented 128 peptides for trypsin (Box A), 61 peptides for pepsin (Box B), 78 peptides for chymotrypsin (Box C) and 36 peptides for papain (Box D). However, differences in peptide population were also observed between fractions obtained from the same enzyme. Pepsin was the enzyme for which this phenomenon was the most pronounced. Indeed, when looking at the heat map, a non-negligible group of peptides was common to the final hydrolysate and the pepsin supernatant but absent from the pellet of the same enzyme. This group represented 41 peptides (Box E) and was completely concentrated in the supernatant following centrifugation. The same phenomenon occurred but on a smaller scale with papain, when centrifugating the hydrolysate obtained with papain; a small group of peptides seemed to be concentrated in the supernatant and less present in the final hydrolysate and pellet (Box F).

#### 3.3.3. Principal Component Analyses (PCA) Scores Report and PCA on Loadings

**Figure 6:**
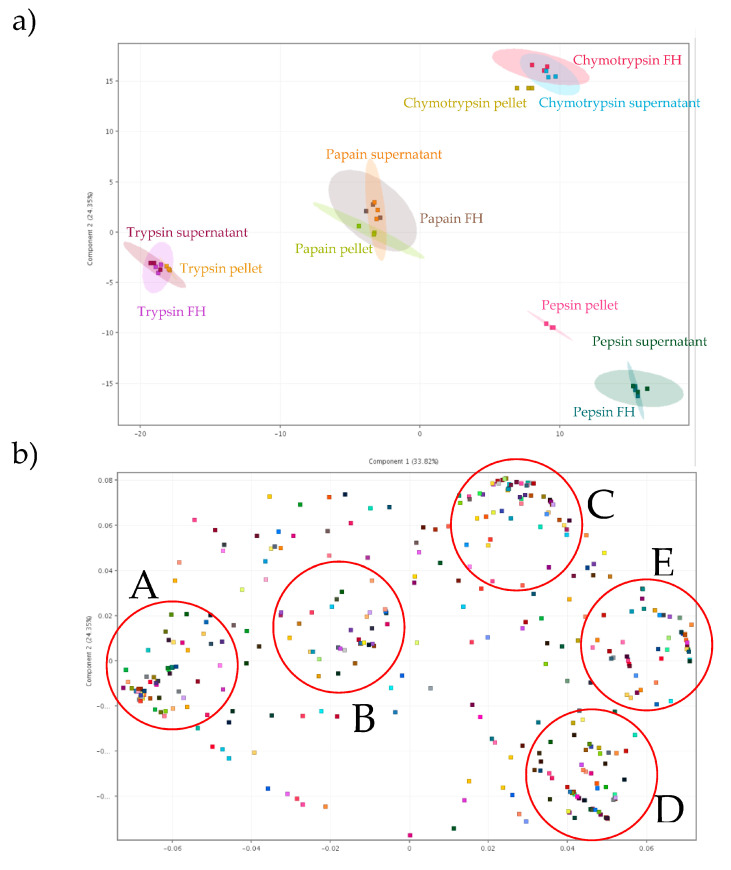
Scatter plots of first principal component (PC) versus second PC, on (**a**) enzyme and fraction conditions (**b**) the 485 peptides, after statistical treatments.

To further investigate the data and elucidate the underlying reasons for those variations, principal component analyses (PCAs) were performed (Figure 6). This statistical method transformed the data into a new coordinate system, allowing variations in the data to be explained with fewer dimensions than the original data [47]. This transformation enabled a better interpretation of the data while preserving a maximum amount of information, as well as allowing graphical visualization of multidimensional data [65]. Initially, a first PCA was conducted on the 12 conditions tested (scores) to statistically assess whether they exhibited discriminative properties. The representation of the three repetitions for each condition was displayed using 95% confidence ellipses (Figure 6a). By representing just two principal components (PCs), 58.17% of the total variance or distribution of variables was specifically accounted for (75.92% with three PCs, data not shown). The variance explained by PC1 (33.82%) was mainly due to trypsin conditions which had the highest negative coordinates on that axis but also to pepsin supernatant and pepsin FH which had the highest positive coordinates on that axis. For other conditions, pepsin pellet and chymotrypsin conditions gathered in the positive part of PC1 while papain conditions gathered in the negative part of the PC1 component. Regarding PC2, the variance could be explained by either chymotrypsin condition and pepsin FH, supernatant and pellet in a lesser degree which had higher positive and negative coordinates, respectively. The 6 other conditions (trypsin and papain conditions) gathered near the 0 of PC2. Conditions using different enzymes were discriminating, while for the fraction type from the same enzyme, only the pepsin pellet appeared different from the other two. Furthermore, the peptides (loadings) were subjected to a second PCA to assess whether statistically significant peptides groups could be identified within the selected population of 485 peptides based on enzymes and fractions conditions (Figure 6b). Five distinct a posteriori groups were highlighted among all recovered and identified peptides (Groups A, B, C, D, E). On PC1, three distinct groups were readily discernible based on their respective positions on this component. In the negative part of this component, two groups were formed (Groups A and B) while another was located in the positive part of this component. Next, when considering PC2, peptides with a positive coordinate on PC1 were further divided into three distinct groups: those with a positive location on PC2 (Group C), those with a negative location on PC2 (Group D) and those whose position gathered around 0 of PC2 (Group E). The two PCAs were compared to gain a more precise interpretation of the formation of these groups. Peptides belonging to group A were found to be influenced by trypsin conditions, peptides from group B were influenced by papain conditions, peptides from group C from chymotrypsin conditions while those belonging to group D were influenced by pepsin conditions. Finally, peptides from group E were more linked to the pepsin conditions and/or the chymotrypsin conditions.

#### 3.3.4. Venn Diagram of Peptides in Final Hydrolysates

**Figure 7:**
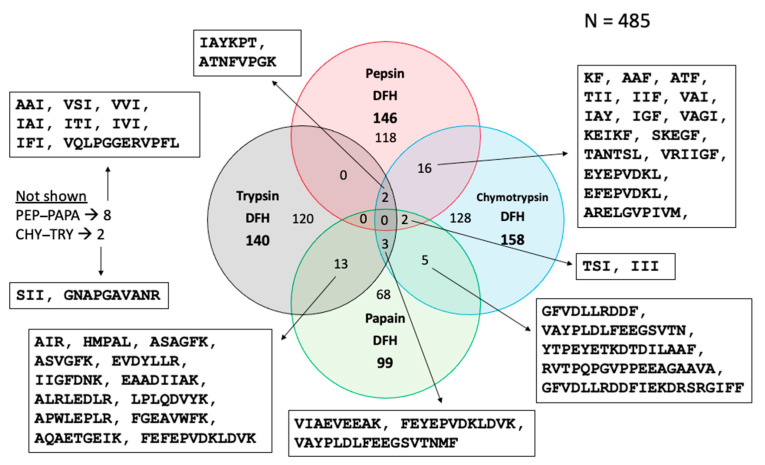
Venn diagram of the peptides in the final hydrolysates (DFH) and sequences common to two or more fractions. PEP: Pepsin, PAPA: Papain, CHY: Chymotrypsin and TRY: Trypsin.

To complete the heat map and PCA information, a Venn diagram showing all possible relationships among the peptide populations found in the hydrolysates (DFH) (*N* = 4) was generated (Figure 7). Of the 485 peptides, 118 were present only in the pepsin hydrolysate, 128 only in the chymotrypsin hydrolysate, 68 only in the papain hydrolysate and 120 only in the trypsin hydrolysate. The lower number of identified sequences in the papain hydrolysate may be explained by its lower DH. This figure shows that most peptides (434) are present only in one fraction, which can be explained by the different specific cleavage sites of each enzyme used. However, there are 44 sequences common to two fractions and 7 common to three fractions since some enzymes have common cleavage sites. No peptide was found to be common to all hydrolysates. To not overcharged the figure, only sequences common to two or more fractions are provided in Figure 7.

### 3.4. Total Phenolic Content (TPC)

**Figure 8:**
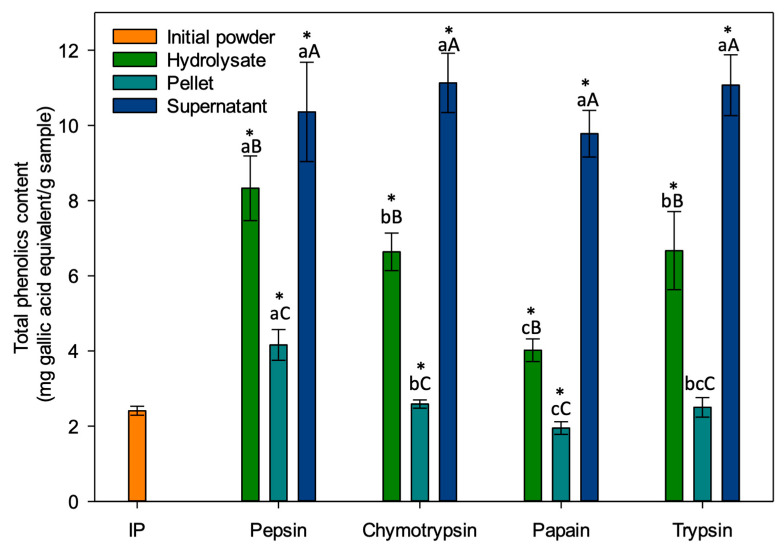
Total phenolic content (TPC) of fractions (*n* = 13). Values are mean ± standard deviation of triplicate. Between fractions of the same enzyme: values with different capital letters are significantly different, according to one-way ANOVA *p* < 0.05 (Tukey test). Between same fraction from different enzymes: values with different lowercase letters are significantly different, according to one-way ANOVA *p* < 0.05 (Tukey test) * An asterisk means a significant difference between the fraction and the initial powder according to *t*-test.

The total phenolic content of the different fractions obtained is presented in Figure 8. Regardless of the enzyme used for hydrolysis, the TPC was always significantly higher in the supernatant (DS) than in the final hydrolysate (DFH) (*p* < 0.001) and the pellet (DP) was the fraction with the lowest total phenolic content (*p* < 0.001). These results were in accordance with Paiva et al., which noted that fractions having a high protein content tend to have a higher TPC [66]. All fractions were significantly different from the initial powder (2.41 ± 0.12 mg gallic acid/g sample) except for the trypsin pellet (2.50 ± 0.26) which showed no significant difference (*p* = 0.585). Rezvankhah et al. obtained similar results when measuring the polyphenols of lentil hydrolysates: TPC was lower for the non-hydrolyzed fraction while the hydrolysates had a significantly higher TPC [67]. They explained this phenomenon by the release, during hydrolysis, of phenolic compounds interacting with proteins by covalent and non-covalent bonds, and also releasing peptides with phenolic groups such as Tyr and Phe [68]. This would explain why IP’s TPC was lower than those of almost all the fractions recovered following hydrolysis.

Also, there was no significant difference in the total phenolic content of the supernatants (*p* = 0.065): The values ranged between 9.78 ± 0.62 and 11.13 ± 0.79 mg gallic acid/g sample. For the final hydrolysates, significant differences were observed (*p* < 0.001): peptic hydrolysate (8.33 ± 1.04) had a higher TPC than chymotryptic hydrolysate (6.64 ± 0.50) and tryptic hydrolysate (6.67 ± 0.86), while the one obtained with papain (4.02 ± 0.30) expressed the lowest content. For the pellets, significant differences were also observed (*p* < 0.001): The pellet obtained with pepsin had the highest TPC (4.16 ± 0.41), followed by the pellet obtained with chymotrypsin (2.59 ± 0.11) and papain (1.95 ± 0.17). The pellet obtained with trypsin was not significantly different from those obtained with chymotrypsin and papain (*p* = 0.948 and *p* = 0.076, respectively). Hernández-Jabalera et al. studied the interaction between peptides and phenolic compounds in rapeseed hydrolysates. They reported that in hydrolysates, phenolic compounds are in a complex form with the polypeptides produced leading to a TPC measurement that is changed according to DH values and variations in TPC values would depend on the types of peptides released, according to their molar mass and amino acid composition [69]. This would therefore explain why a positive relationship between TPC and DH was observed in the present study, i.e., higher TPC when DH was higher for hydrolysates and pellets.

Only few studies have investigated TPC in duckweeds but without hydrolysis. A first group of researchers evaluated TPC of *Wolffia arrhiza* and obtained 7.57 ± 0.26 mg gallic acid equivalent/g dry weight [46], which is higher than the result for initial powder used in this study (~2.4 mg gallic acid equivalent/g) but lower than the result for supernatant fractions (between 9.78 and 11.13 mg gallic acid/g sample). In their study, they triply extracted the samples using ethanol 95% as solvent at 50 °C for 30 min at a 1:30 (*w*/*v*) ratio and they centrifuged after extraction to measure TPC on supernatant only. In contrast, in the present study, the extraction was carried out only once using methanol 80% as solvent at 50 °C for 60 min at a concentration of 25 mg/mL in an ultrasonic bath and the solutions filtered to remain consistent in the methodology (samples filtered for OPA and RP-UPLC analyses as well). There were also differences in the method used to determine the TPC: the experiments were performed in a microplate whereas the authors did not, and the incubation time before reading was 30 min in the present case whereas it was one hour in theirs. Differences in the methodology used and in the raw material (*Wolffia arrhiza* vs. a blend of duckweed species) may explain these different results [21,70,71,72]. A second group of researchers assessed TPC of *Wolffia globosa* and obtained a value of 40.83 ± 4.99 mg Gallic Acid Equivalent/g extract [22]. This result was almost 4 times higher than the highest TPC value in this study. In their study, the authors started with raw duckweed, washed it, boiled it for 15 min and dried it at 50 °C while no information was available according to the pre-treatment of the product in the present study since a blend of duckweed species in powder form was used. Then, they extracted it twice for 7 days using ethanol 95%, while in the present study the extraction was carried out once for 1 h at 50 °C in an ultrasonic bath using methanol 80%. Finally, the authors filtered the extracts as in this study, but they evaporated the extracts and froze them to assess TPC later using Folin-Ciocalteu method (same method) as in the present study, dosage of phenolic compounds was immediately measured after the extraction. For dosage of TPC, the samples were prepared at a concentration of 1 mg of extract in 1 mL of ethanol which was four times more concentrated than what was done in the present study. Their higher sample concentrations were therefore the main element explaining the differences in TPC results.

Since plants are known to have a high polyphenol content and polyphenols are known to exhibit antihypertensive activity [61,66], it was important to measure its content in the various fractions to be able to discriminate whether antihypertensive activity is attributable to peptides only.

### 3.5. Evaluation of Antihypertensive Activity

#### 3.5.1. Evaluation of ACE-Inhibition

In this study, the antihypertensive activity was assessed through the inhibition of ACE, an enzyme responsible for converting angiotensin I into angiotensin II, thereby triggering an elevation in blood pressure. This evaluation is quantified by the IC_50_ value, denoting the concentration required to inhibit 50% of the enzyme’s activity. Consequently, a fraction demonstrating a lower IC_50_ is of greater interest for its antihypertensive activity since a reduced concentration is needed for effective enzyme inhibition.

**Figure 9:**
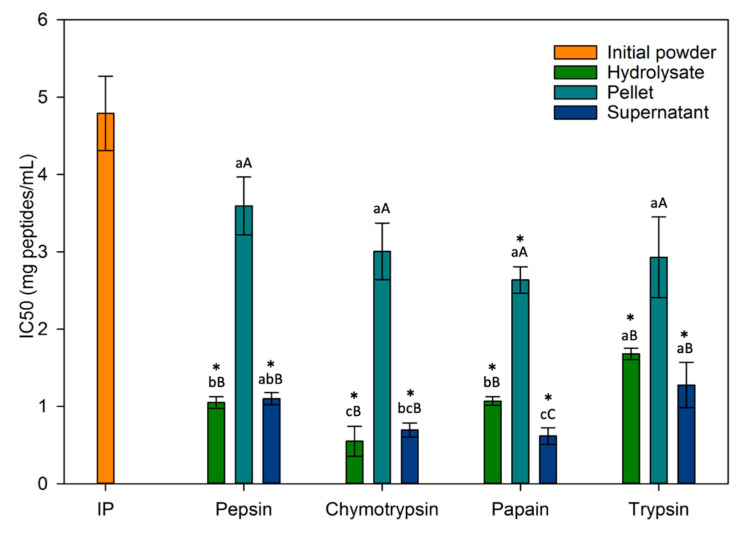
IC_50_ values of fractions (*n* = 13). Values are mean ± standard deviation of triplicate. Between fractions of the same enzyme: values with different capital letters are significantly different, according to one-way ANOVA *p* < 0.05 (Tukey test). Between same fraction from different enzymes: values with different lowercase letters are significantly different, according to one-way ANOVA *p* < 0.05 (Tukey test). * An asterisk means a significant difference between the fraction and the initial powder according to *t*-test.

First of all, Figure 9 shows that the ACE-inhibitory activity of hydrolysates and supernatants were significantly higher (*p* < 0.05) (lower IC_50_ values between 0.55 ± 0.19 and 1.68 ± 0.07 mg peptides/mL) when compared to the non-hydrolyzed initial powder (IC_50_ = 4.79 ± 0.48 mg peptides/mL) which can be attributed to a higher peptide content and/or TPC in these fractions than in the initial powder.

Due to limited available literature on ACE inhibition of duckweed protein hydrolysates/fractions, it was challenging to make a meaningful comparison between the current data and other findings. However, the IC_50_ values obtained in this study for hydrolysate and supernatant fractions (between 0.55 ± 0.19 and 1.68 ± 0.07 mg peptides/mL) were lower than that obtained for eggplant leaf protein hydrolysates (supernatants) which were between 2.1 and 2.7 mg peptides/mL [34]. Some fractions (chymotrypsin hydrolysate, chymotrypsin supernatant and papain supernatant) were even lower than the purified fractions in their study which ranges between 0.81 and 0.83 mg peptides/mL demonstrating the antihypertensive potential of certain peptides in our fractions. On amaranth leaf protein hydrolysates hydrolyzed by alcalase, trypsin, pepsin and chymotrypsin, Famuwagun et al. obtained IC_50_ values between 0.29 and 0.97 mg/mL depending on the enzyme used [13], results that were similar to this study (between 0.55 ± 0.19 and 1.68 ± 0.07 mg peptides/mL). Once again, these tests were only performed on the supernatants. The peptic hydrolysate was the most antihypertensive in their study, while the fraction from hydrolysis with chymotrypsin was the least antihypertensive. In the present study, supernatants from hydrolysis with chymotrypsin and papain were the most antihypertensive, while those from trypsin and pepsin were the least interesting. This shows that an enzyme’s ability to produce antihypertensive peptides depends on the protein source hydrolyzed, i.e., its amino acid content. Paiva et al. assessed ACE-inhibitory activity of *Fucus spiralis* hydrolysates (by cellulase and bromelain) and its fractionations by ultrafiltration. Even if the ACE-inhibition was not determined with the same method (HPLC-UV method in their study and spectrophotometric assay in this study), their IC_50_ values between 0.500–2.000 mg/mL were similar to those obtained in this study for hydrolysates and supernatant between 0.5 and 1.7 mg/mL [66]. It was important to note that the fractions from the present study have not even been fractionated compared to those from Paiva et al. and Famuwagun et al., demonstrating once again the high antihypertensive potential of fractions.

However, most studies assessing ACE-inhibition, did not measure the TPC and, as indicated previously and pointed out by others, plants are known to present high polyphenol contents known to exhibit antihypertensive activity [61,66]. Consequently, most of the time, it was not possible to discriminate whether antihypertensive activity was attributable to peptides only, to a synergistic effect between polyphenols and peptides or to peptides with phenolic groups. In the present study, it could be highlighted from both TPC and ACE-inhibitory activity data that for PEP fractions, TRY fractions, CHY DP and PAPA DP, the TPC (in comparison with the IP taken as a control) increased of approximately the same fold as the ACE inhibitory activity (IP taken as a control) (Table 3) and this suggested mainly an effect of the TPC on the ACE-inhibitory activity due to the release of phenolic compounds during hydrolysis [66]. In contrary, for CHY DFH, CHY DS, PAPA DFH and PAPA DS, the ACE inhibitory activity increased by 8.7, 6.8, 4.5 and 7.7, respectively while TPC only by 2.8, 4.6, 1.7 and 4.1, respectively (values in bold in Table 3): this suggested that the ACE inhibitory activity would be mainly due to peptides present or a synergistic effect between polyphenols and specific peptides released during these hydrolyses.

Furthermore, it was interesting to note that for all enzymes except papain, centrifugation and recovery of the supernatant only, did not increase the IC_50_ value compared to hydrolysate, which raised the question of the relevance of this step to enhance the biological activity being studied. It appeared that the most efficient fractions were the ones obtained by hydrolysis of the whole duckweed powder. Additionally, non-protein compounds did not disappear during hydrolysis and the different fractions obtained necessarily contained compounds other than peptides or proteins such as soluble and insoluble fibers, phenolic compounds, and ashes [34]. Thus, when compared to chymotrypsin and trypsin supernatants, pepsin and papain’s supernatant lower protein contents indicated the existence of higher concentrations of other compounds, which may have an influence (positively or negatively) on their biological activities [61] and not only on antihypertensive activity.

#### 3.5.2. Screening for Potential Antihypertensive Sequences

Among sequences given in Figure 7, KF, ATF and IAY, all sequences common to pepsin and chymotrypsin hydrolysates were found to be potential antihypertensives when entering them in HTPDB: Database of Antihypertensive Peptides [73]. Information about the sequences, namely source, IC_50_ values and reference article are provided in Table 4.

## 4. Conclusions

The results presented in this study revealed that duckweed powder was efficiently hydrolyzed by pepsin, chymotrypsin, papain and trypsin, demonstrated by DH values ranging from approximately 3% to 9%, even without prior extraction and concentration of proteins. While most studies involving enzymatic hydrolysis of proteins primarily focus on the hydrolysate supernatant, the protocol carried out in this study enabled the recovery and study of three distinct fractions (DFH, DS and DP) for each hydrolysis. The protein/peptide content and recovery yield of each fraction were contingent upon the degree of hydrolysis. Moreover, peptides present in the various fractions were characterized and identified using UPLC-MS/MS, unveiling differences in peptide populations between fractions derived from different enzymes, as well as between diverse fractions originating from the same enzyme. This underscored that the centrifugation step enabled the concentration of specific peptides within certain fractions. Overall, most of the peptides identified in this work (485) were unique to a specific hydrolysate, while 51 peptides were common to two or three hydrolysates. Evaluation of TPC indicated no significant differences between the four supernatants (DSs). Regarding the final hydrolysates (DFH), it was noted that the highest polyphenol content did not necessarily correspond to the highest antihypertensive activity. These findings suggest that the nature of the peptides present in the fractions could be responsible for the antihypertensive effect. The chymotryptic final hydrolysate (DFH), the chymotryptic supernatant (DS) and the papain supernatant (DS) emerged as the most interesting fractions in terms of antihypertensive activity within this study, with IC_50_ values between 0.55 and 0.70 mg peptides/mL. The paucity of studies on the enzymatic hydrolysis of duckweed highlighted the relevance of this study. To our knowledge, this was the first study to investigate the enzymatic hydrolysis of duckweed proteins to produce bioactive peptides with therapeutic applications in mind.

Currently, a more comprehensive investigation into the 485 identified sequences is in progress to identify the peptide sequences responsible for biological activities. This will involve a comparison between peptides retrieved from hydrolysis and centrifugation fractions and those from the non-centrifugated fraction. Furthermore, several characteristics of the identified peptides will be closely examined, including molecular weight, charge and amino acid composition, aiming to identify potential antihypertensives. Additionally, the different fractions will be evaluated in vitro for other biological activities, such as anti-diabetic and antioxidant activities. Looking ahead, as a prospective avenue, once the peptides of interest would be determined, two possibilities may be contemplated; (1) to synthesize the peptides and to test them individually to assess their effective biological activity, and (2) a purification step to concentrate these peptides, thereby increasing their biological activity(ies).

## Figures and Tables

**Table 1:** Number of theoretical cleavage sites for each enzyme, number of identified peptides originating from this protein and proportion of the total number of sequences identified.

Enzyme	Number of Cleavage Sites	Number of Identified Peptides Derived from this Protein/Number of Total Identified Peptides	Proportion of the Total Number of Sequences Identified (%)
Pepsin	112	46/146	31.5
Chymotrypsin	100	57/158	36.1
Papain	141	42/99	42.4
Trypsin	48	32/140	22.9

**Table 2:** Protein/peptide content and protein/peptide recovery yield of the different fractions.

Enzyme	Protein/Peptide Content (%)			Protein/Peptide Recovery Yield (%)	
	DFH	DP	DS	DP	DS
Pepsin	39.14 ± 1.02 ^aB^*	26.34 ± 0.89 ^bcC^	50.12 ± 0.65 ^dA^	40.43 ± 2.88 ^bB^	51.24 ± 5.11 ^aA^
Chymotrypsin	40.01 ± 2.33 ^aB^	27.02 ± 1.93 ^bC^	64.73 ± 0.95 ^bA^	48.34 ± 3.15 ^bA^	46.30 ± 8.57 ^aA^
Papain	36.16 ± 2.55 ^aB^	34.84 ± 0.15 ^aB^	56.93 ± 1.29 ^cA^	80.06 ± 5.91 ^aA^	27.13 ± 1.61 ^bB^
Trypsin	40.47 ± 3.08 ^aB^	23.86 ± 0.42 ^cC^	67.77 ± 1.13 ^aA^	41.43 ± 3.40 ^bB^	50.44 ± 4.07 ^aA^

* Values are mean ± standard deviation of triplicate. Column-wise: values with different lowercase letters are significantly different, according to one-way ANOVA *p* < 0.05 (Tukey test for purity and *t*-test for recovery yield). In rows: values with different capital letters are significantly different, according to one-way ANOVA *p* < 0.05 (Tukey test).

**Table 3:** Fold-increase protein/peptide content, TPC and ACE inhibition activity of fractions in relation to IP.

	Protein/Peptide Content	TPC	ACE
IP	1.00	1.00	1.00
PEP DFH	1.00	3.46	4.56
PEP DP	0.67	2.49	1.33
PEP DS	1.28	4.30	4.35
CHY DFH	1.00	**2.76**	**8.71**
CHY DP	0.68	1.07	1.60
CHY DS	1.62	**4.62**	**6.84**
PAPA DFH	1.00	**1.67**	**4.48**
PAPA DP	0.96	0.81	1.81
PAPA DS	1.57	**4.06**	**7.73**
TRY DFH	1.00	2.77	2.85
TRY DP	0.59	1.04	1.63
TRY DS	1.67	4.59	3.74

**Table 4:** Sequences common to two or more hydrolysates with antihypertensive potential.

Sequence	Source	IC50	Reference
KF	Wakame	28.3 μM	Suetsuna et al. (2004) [74]
ATF	Cereals (Barley protein B-Hordein)	9.6 μM	Gu et al. (2011) [75]
IAY	ND	<20 mM	Wu et al. (2006) [76]

## Data Availability

The data presented in this study are available on request from the corresponding author. The data are not publicly available due to ongoing industrial collaborative project.
